# Potential Resistance to Antineoplastic Aminated Fullerenes Mediated by M2-Like Monocyte-Derived Exosomes

**DOI:** 10.3389/fonc.2022.779939

**Published:** 2022-03-31

**Authors:** Jiawei Huo, Wei Zhou, Yang Liu, Sifen Yang, Jie Li, Chunru Wang

**Affiliations:** ^1^ Beijing National Research Center for Molecular Sciences, Key Laboratory of Molecular Nanostructure and Nanotechnology, Institute of Chemistry, Chinese Academy of Science, Beijing, China; ^2^ University of Chinese Academy of Sciences, Beijing, China

**Keywords:** C70-EDA, M2-like monocyte-derived exosome, proteomics, Rho GTPase/PAK signaling, tumor proliferation

## Abstract

Exosomes are small extracellular vesicles critical for intercellular signaling *via* their delivery of cargoes, including proteins, DNA, RNA, lipids, and metabolites. Exosomes play essential roles in remodeling the tumor microenvironment (TME) for tumor growth, metastasis, and drug resistance. Aminated fullerenes (e.g., C_70_-ethylenediamine [EDA]) exhibit antineoplastic effects by targeting multiple functional proteins. Nanosized C_70_-EDA with positive surface charges tends to be taken up by monocytes in the bloodstream and monocyte-derived macrophages in the TME. Herein, the alterations of monocytes and monocyte-derived exosomes by C_70_-EDA have been investigated. C_70_-EDA reprogramed THP-1 monocyte to an M2-like state and substantially increased the protein content in exosomes secreted by M2-like monocytes. Notably, C_70_-EDA-induced M2-like monocytes released exosomes that triggered the proliferation of recipient tumor cells, which may alleviate the antineoplastic efficacy of C_70_-EDA. As revealed by proteomic profiling of exosomes, this outcome is probably a result of Rho GTPase/p21-activated kinase (PAK) pathway activation in recipient tumor cells induced by upregulated exosomal proteins. This work indicates a promising strategy in which aminated fullerenes can be combined with PAK inhibitors for cancer therapy.

## Introduction

Exosomes with a size range of ~40 to 160 nm in diameter constitute a subset of extracellular vesicles of endosomal origin and are generated through the fusion and exocytosis of multivesicular bodies. Exosomes play fundamental roles in intercellular communication and regulation *via* the delivery of diverse cargoes (cytoplasmic and cell surface proteins, DNA, RNA, lipids, and metabolites) from exosome-secreting cells to recipient cells (1–5). Exosomes derived from different cell sources exhibit diverse functions in regulating tumor immunity, ischemic diseases, angiogenesis, and hepatic pathology (6–9). In tumor therapy, exosomes can mediate drug efflux, prosurvival signaling, and tumor microenvironment (TME) remodeling (10–12), resulting in resistance to chemotherapy, targeted therapy, and immunotherapy (13–15).

Monocytes are innate immune cells that circulate in the bloodstream and can be recruited throughout tumor progression. Recently, monocytes have emerged as essential regulators of tumorigenesis and metastasis. In response to diverse stimuli, different monocyte subsets can even perform opposing roles in protumoral and antitumoral immunity, including phagocytosis, secretion of tumoricidal mediators, and differentiation into tumor-associated macrophages (TAMs) (16). Monocyte-derived TAMs can be further polarized into one of two phenotypes (M1 and M2) (17), in which M1 exhibits cytotoxic (antitumoral) effects and M2 plays protumoral roles (18). M2 macrophage-derived exosomes can regulate cancer proliferation and metastasis, promote angiogenesis, and generate drug resistance (19, 20). Functional proteins enriched in M2 macrophage-derived exosomes are shuttled to tumor cells to promote tumor progression *via* diverse mechanisms. For example, increased arginase-1 (an enzyme that converts arginine to ornithine and urea) in M2 macrophage-derived exosomes is critical for glioblastoma cell proliferation (21), and exosomal CD11b/CD18 and apolipoprotein E (ApoE) separately activate the MMP-9 and PI3K/AKT pathways in recipient tumor cells to promote their migration (20, 22).

Functional fullerene derivatives exhibit notable antineoplastic effects and a wide safety margin (23–25). Hydroxylated fullerenes inhibit tumor growth and metastasis by attenuating the TME through, for example, reactive oxygen species scavenging, immune modulation, and angiogenesis inhibition (26). Compared with hydroxylated fullerenes, aminated fullerenes (e.g., C_70_-ethylenediamine [EDA]) can target multiple functional proteins and directly abrogate tumor cell proliferation and migration (27, 28). Nanosized fullerene derivatives tend to be taken up by TAMs and subsequently induce TAM polarization to the M1 subtype, making the TME susceptible to tumor therapy (29, 30). Aminated fullerenes with clarified targets have potential applications in targeted therapies. However, further study is required to determine their roles in shaping the TME *via* alterations of monocytes and monocyte-derived exosomes. This study reveals that C_70_-EDA indeed shapes THP-1 monocyte to an M2-like state (monocyte with features of M2 macrophage) and significantly enhances the protein contents in M2-like monocyte-derived exosomes. Moreover, the exosomes boost recipient tumor cell proliferation, probably by activating the Rho GTPase/p21-activated kinase (PAK) pathway.

## Materials And Methods

### Materials

C_70_-EDA was prepared, characterized, and fluorescently labeled with FITC (abbreviated C_70_-EDA-FITC) as described in our previous work (27). All commercial reagents were used without further purification.

### Cell Culture and Treatment

THP-1 human monocytes, A549 human lung cancer, and U87-MG human glioma tumor cell lines were purchased from the Cell Resource Center of Peking Union Medical College. THP-1 cells were cultured in RPMI-1640 (Mediatech, Manassas, VA, USA, Cat. #10-040, RPMI-1640 with L-glutamine) medium containing 10% exosome-depleted fetal bovine serum (VivaCell Biosciences, Shanghai, China, Cat. #C3801-0100) and 1% penicillin/streptomycin (Gibco, Waltham, MA, USA, Cat. #15140122). A549 and U87-MG cells were cultured in Dulbecco’s modified Eagle’s medium (DMEM) (Mediatech, Manassas, VA, USA, Cat. #10-013, 4.5 g/L glucose, L-glutamine, and sodium pyruvate) containing 10% exosome-depleted fetal bovine serum (VivaCell Biosciences, Shanghai, China, Cat. #C3801-0100) and 1% penicillin/streptomycin (Gibco, Waltham, MA, USA, Cat. #15140122) in a 5% CO_2_ incubator at 37°C.

### Exosome Isolation

THP-1 cells were plated at a density of 100,000 per milliliter in the 75 cm^2^ cell culture flask (Corning, NY, USA, Cat. #353135) and cultured for 48 h. For vesicle enrichment, the culture medium was centrifuged at 300 × g for 10 min at room temperature, followed by 2,000 × g for 20 min at 4°C and then 12,000 × g for 40 min at 4°C (Allegra X-30R Centrifuge, Beckman Coulter Inc., Brea, CA, USA). The supernatant was then centrifuged twice at 100,000 × g for 1 h and then 10 min at 4°C for exosome purification (OPTIMA L100XP, Beckman Coulter Inc., Brea, CA, USA).

### Nanoparticle Tracking Analysis (NTA)

Exosomes were diluted (1/5) in PBS and measured by NTA to determine the concentration and size distribution using the particle-matrix ZetaView PMX 110 multiple parameters particle tracking analyzer (Particle Metrix, Meerbusch, Germany) with 405 nm emitted light.

### Transmission Electron Microscopy (TEM)

Transmission Electron Microscopy (TEM) was performed on isolated exosomes after fixation on a 400-mesh square copper grid with 2% uranyl acetate using a negative staining method. A copper net was dripped with 10 µL of exosome solution, incubated for 10 min at room temperature, and then washed with sterile distilled water. Excess liquid was removed with blotting paper. Then, for negative staining, 10 µL of 2% uranyl acetate dihydrate was dripped onto the copper net for 1 min. The floating solution was removed with filter paper, and the sample was dried under incandescent light for 2 min. The copper net was observed and imaged at 80 kV with an HT7700 transmission electron microscope (HITACHI, Tokyo, Japan).

### MALDI-TOF-MS

Matrix-assisted laser desorption/ionization-time of flight-mass spectrometry (MALDI-TOF-MS) was performed with a Bruker Autoflex-III Smart Beam (Bruker Daltonics, Bremen, Germany). Cyano-4-hydroxycinnamic acid (CHCA) was dissolved in acetonitrile to form a matrix (15 mg/mL). An aqueous solution of fullerene derivatives was mixed with the same volume of matrix solution, and then 1 μL of the combined solution was transferred to the stainless steel target. The target dried at room temperature was loaded into the ion source for analysis. The mass spectra were obtained with the Smart Beam laser (355 nm) operating at 100 Hz with a laser focus of 50 μm. The plate offset voltage was set to 19 kV, and the deflection detector voltage was set to 20 kV. The data were processed with Data Analysis 3.0 software (Bruker Daltonics, Bremen, Germany).

### Ultraviolet-Visible (UV–vis) Absorption Spectroscopy

The UV–vis absorption spectra of THP-1 exosomes, C_70_-EDA, and THP-1 exosomes isolated after C_70_-EDA treatment and in aqueous solution were acquired with a UH4150 UV–Visible/NIR spectrophotometer, produced by Hitachi High Technologies Corporation (Tokyo, Japan), with a scan range from 200 to 800 nm.

### Antibodies

Anti-iNOS antibody (Cat. #ab178945), anti-NF-κB p65 antibody (Cat. #ab32536), anti-IL-12 p40 antibody (Cat. #ab131156), anti-CD163 antibody (Cat. #ab182422), and anti-CDC42 antibody (Cat. #ab187643) were purchased from Abcam (Cambridge, MA, USA). Anti-β-actin antibody was purchased from Cell Signaling Technology (Danvers, MA, USA), (Cat. #4970); RAC1 polyclonal antibody (Cat. #24072-1-AP) was purchased from Proteintech (Chicago, IL, USA). Anti-rabbit IgG, HRP-linked antibody was purchased from Cell Signaling Technology (Danvers, MA, USA), (Cat. #7074) as the secondary antibody for WB analysis. All primary antibodies were sourced from rabbits and used at a 1:1,000 dilution for western blot (WB) assays.

### *In Vitro* Cytotoxicity

A Cell Counting Kit-8 (CCK-8) assay was used to test the cytotoxicity induced by C_70_-EDA in THP-1 cells. Cells were cultured in 96-well plates for 24 h and incubated for 24 h with C_70_-EDA at concentrations from 0 to 200 μM in the dark at 37°C, and then, cell viability was detected by Cell Counting Kit-8 (CCK-8, DOJINDO, Kumamoto, Japan). The absorption value (optical density [OD]) of the CCK-8 reagent measured at 450 nm was read with a 96-well plate reader (iMark microplate reader, Bio-Rad, Hercules, CA, USA) to determine the viability [cell viability = (ODtre − ODmedium)/(ODcon − ODmedium)], where ODtre is the absorption of the treated cells read at 450 nm, ODcon is that of the control cells, and ODmedium is that of the culture medium.

### The Coculture System

THP-1 cells were cocultured with A549 cells in a Transwell cell culture. Briefly, THP-1 cells were pretreated with C_70_-EDA (20 μM) for 48 h, seeded in 24-well plates at a density of 5 × 10^5^ cells/well and then cocultured with A549 cells seeded in the upper chamber (1 × 10^4^/well) of Corning Costar Transwell 3422 culture plates for 24 h.

### Western Blotting

The gel and immunoblot reagents were purchased from Bio-Rad (Hercules, CA, USA). Cells or exosomes were lysed in RIPA buffer, and the cellular or exosomal protein concentration was determined using a bicinchoninic acid (BCA) protein quantification kit (Beyotime, Jiangsu, China, Cat #P0012). On the basis of the quantification results, the appropriate amount of 5X SDS buffer was added, vortexed, and mixed, and electrophoretic separation of the proteins was performed after denaturation at 95°C for 5 min. Equal amounts of protein extracts (30 μg) were loaded onto each well of a 4-20% Mini-Protean TGX precast gel (Bio-Rad, Hercules, CA, USA). After gel electrophoresis, the proteins were transferred from the gel to polyvinylidene fluoride membranes (0.45 μm, Millipore, Burlington, MA, USA). Subsequently, the membranes were blocked with 5% nonfat milk at 4°C for 1 h under agitation. Following rinsing, the membranes were incubated with primary antibodies overnight. After washing three times with TBST, we incubated the membranes with the secondary antibody for 1 h at 4°C. Finally, the proteins were visualized by enhanced chemiluminescence (ECL) detection reagents (Millipore, Burlington, MA, USA) and quantified with a Gel Image system, ver. 4.00 (Tanon, Shanghai, China). Primary and secondary antibodies were used at dilutions of 1:1,000 and 1:5,000, respectively. β-Actin was used as the reference protein for quantification.

### RT–PCR Assay

Total RNA was extracted from cultured cells or exosomes using TRIzol S3 lysate (Invitrogen Life Technologies, Carlsbad, CA, USA, Cat. #15596-026), and RNA (1 μg) was converted to cDNA with a 1st Strand cDNA Synthesis SuperMix kit (NovoScript, Suzhou, China, Cat. #E044-01A). Then, fluorescence (FL) quantification was performed using an SYBR One-Step qRT–PCR Kit (NovoScript, Suzhou, China, Cat. #E092-01A) in a 20 μL reaction volume. Real-time PCR was performed with an ABI 7500 fast system as previously described. Glyceraldehyde-3-phosphate dehydrogenase (GAPDH) was the reference gene for quantification, and the primer sequences are listed in the supporting information (**Table S1**).

### Confocal FL Imaging

THP-1 cells were seeded in confocal plates at a density of 5000 cells/mL and incubated at 37°C in 5% CO_2_ for 24 h. The cells were treated with 20 μM C_70_-EDA-FITC for 24 h. The cells were treated with 100 nM DiD (Beyotime, Jiangsu, China, Cat #C1039) for 30 min in the dark. Confocal images were captured using a fluorescence microscope system (FV1000, Olympus, Tokyo, Japan) under the following conditions: The FITC tag was excited at 488 nm, and the FL was recorded from 500 to 545 nm; DiD was excited at 559 nm, and the FL was recorded from 570 to 670 nm.

### Proteomic Profiling of Exosomes

Exosomes were isolated from THP-1 cells treated with 20 μM C_70_-EDA for 24 h. Exosomal proteins were extracted, quantified, and then enzymatically digested for label-free protein mass spectrometry detection. The obtained MS data were searched in the MaxQuant software database for protein identification and subsequent bioinformatics analysis. Proteomics analysis of exosomes was performed with the assistance of Echo Biotech, China. In brief, the first step is to identify the peptide signal in each LC-MS data, obtain the molecular weight information of the corresponding peptide of the protein by theoretical enzymatic cleavage, perform a search of the primary parent ion, and then perform a database search of the MS2 of all peptide signals to obtain the corroboration of the exact secondary sequence, and finally integrate all the qualitative and quantitative data. The limma R package was used to analyze the between-sample differences, using |log2(FC)|≥1, FDR ≤ 0.01 as the screening criteria to detect differentially expressed proteins. Fold Change (FC) indicates the expression ratio between two samples (groups). The significance p-value (p-value) obtained from the original hypothesis could be expressed as the probability of no difference in expression. Afterward, the proteins were functionally annotated using NCBI Non-Redundant database (NR), Gene Ontology (GO), clusters of orthologous groups (COG), Kyoto Encyclopedia of Genes and Genomes (KEGG), and clusters of euKaryotic Orthologous Groups (KOG) databases, enriched for differential proteins between sample groups using topGO software, and annotated for protein structural domains using the Pfam database. Finally, the differentially expressed proteins in the analysis were combined with the reciprocal relationship pairs in the STRING database to construct the differentially expressed protein network. The images were plotted using Cytoscape software.

### Statistical Analysis

All the data are presented as the means ± standard deviation (SD). Statistical analysis was performed with GraphPad Prism 8.2 software (GraphPad Software, San Diego, CA, USA). The results were analyzed using ANOVA or Student’s t-test. Differences were considered to be statistically significant when P < 0.05.

## Results

### Alteration of THP-1 Monocytes to M2-Like State by C_70_-EDA

**Figure 1:**
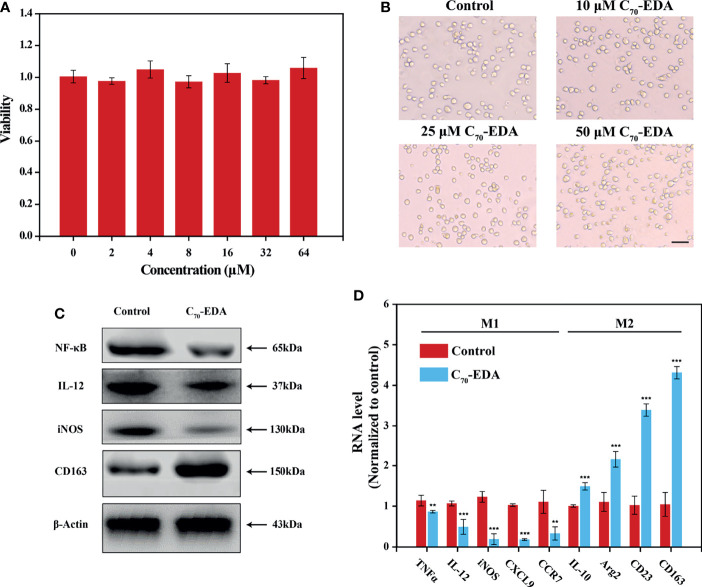
C_70_-EDA induced THP-1 cells to an M2-like state. **(A)** Viability and **(B)** morphology of THP-1 cells treated with different concentrations of C_70_-EDA for 24 h (n = 4). The scale bar is 20 μm. **(C, D)** THP-1 cells were treated with PBS (control) or 50 μM C_70_-EDA for 24 h. **(C)** The protein levels of M1 markers (NF-κB, IL-12, and iNOS) and M2 markers (CD163) in THP-1 cells as determined by Western blotting. **(D)** The mRNA levels of M1 markers (TNFα, IL-12, iNOS, CXCL9, and CCR7) and M2 markers (IL-10, Arg2, CD23, and CD163) by RT–PCR (n = 4). The data are reported as the means ± s.d., and n represents the number of biologically independent samples. Statistical significance was calculated by Student’s t-test. **p < 0.01 and ***p < 0.001.

C_70_-EDA was prepared and characterized as reported previously (27). The half-maximal inhibitory concentration (IC_50_) of C_70_-EDA in tumor cells is approximately 20 μM. THP-1 monocyte is a standard model to investigate monocyte-macrophage differentiation. In this work, the viability and morphology of THP-1 cells were not changed by C_70_-EDA at concentrations up to 64 μM (**Figures 1A, B** **)**. Western blotting and RT–PCR assays were performed to assess the expression of typical protein markers and immune cytokines in THP-1 cells. After C_70_-EDA treatment, the protein level of CD163 increased, and that of IL-12 and iNOS decreased (**Figure 1C**). These findings indicated that C_70_-EDA induced THP-1 cells to exhibit the features of M2 macrophage. Additionally, the expression of typical factors of M1 macrophage (TNFα, IL-12, iNOS, CXCL9, and CCR7) was downregulated, whereas that of M2 macrophage (IL-10, Arg2, CD23, and CD163) was upregulated (**Figure 1D**). C_70_-EDA treatment still maintains the original morphology and adherent state of THP-1 cells, indicating that C_70_-EDA did not differentiate THP-1 monocytes to macrophages. Interestingly, THP-1 monocytes treated by C_70_-EDA indeed exhibited unique features of M2 macrophage, including the up-regulation of M2 markers and the corresponding down-regulation of M1 markers. To avoid misleading, we refer to the M2 phenotype of THP-1 monocyte induced by C_70_-EDA as M2-like monocyte, and analogous monocyte phenotype and nomenclature have been reported (31–33). Fullerene derivatives are thought to induce TAM polarization by regulating NF-κB expression (34). C_70_-EDA reduced NF-κB expression in THP-1 cells (**Figure 1C**), probably resulting in the M2-like conversion of monocytes.

### Isolation and Identification of Exosomes

**Figure 2:**
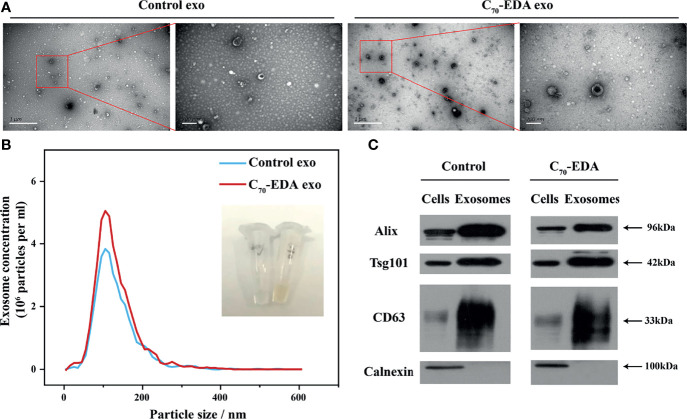
Characterization of exosomes secreted by THP-1 cells treated with C_70_-EDA (20 μM). **(A)** TEM images of the exosomes. Scale bars are 1 μm (left) and 200 nm (right). **(B)** Hydrodynamic diameter distribution and optical image of the exosomes. **(C)** Expression of specific markers (Alix, TSG101, CD63, and calnexin) in the exosomes. Alix, apoptosis-linked gene-2-interacting protein X; TSG101, tumor susceptibility gene 101.

Exosomes secreted by THP-1 cells treated with C_70_-EDA (20 μM) were collected *via* ultracentrifugation isolation and abbreviated as C_70_-EDA exosomes. As shown in the TEM images, small membrane vesicles with a diameter of approximately 100 nm and a typical exosomal cup-like structure were observed (**Figure 2A**), indicating that C_70_-EDA treatment did not affect the morphology or size of the exosomes. As revealed by NTA, the hydrodynamic diameter of both the control exosomes and C_70_-EDA exosomes was approximately 100 nm, and the concentration of both exosome types was 4×10^6^ exosomes/mL (**Figure 2B**). The expression of exosomal biomarkers (CD63, CD81, TSG101, and Alix) was upregulated in the control exosomes and C_70_-EDA exosomes, and a negative marker (calnexin) was not expressed in these exosomes (**Figure 2C**), confirming the successful isolation of exosomes.

The protein content of the C_70_-EDA exosomes (422.83 mg/L) was three times higher than that of the control exosomes (121.6 mg/L), indicating that an increase in exosomal proteins was induced by C_70_-EDA treatment. In addition, the C_70_-EDA exosomes had a yellow color and UV–vis absorption spectra similar to those of C_70_-EDA (**Figures 2B** and **S1A**), and the characteristic mass signal of the fullerene carbon cage (C_70_, m/z: 840) was detected in the C_70_-EDA exosomes (**Figure S1B**), revealing that C_70_-EDA entered the exosomes. In addition, THP-1 cells were treated with fluorescently labeled C_70_-EDA (C_70_-EDA-FITC), and the cell membrane was stained with DiD. The FL of C_70_-EDA-FITC and DiD in the cells mostly overlapped (**Figure S2**), confirming the uptake of C_70_-EDA by THP-1 cells. In conclusion, C_70_-EDA treatment led to an increase in the protein contents of exosomes secreted by THP-1 cells and entered the exosomes without changing their morphology or yield.

### Proteomic Profiling of Exosomes

**Figure 3:**
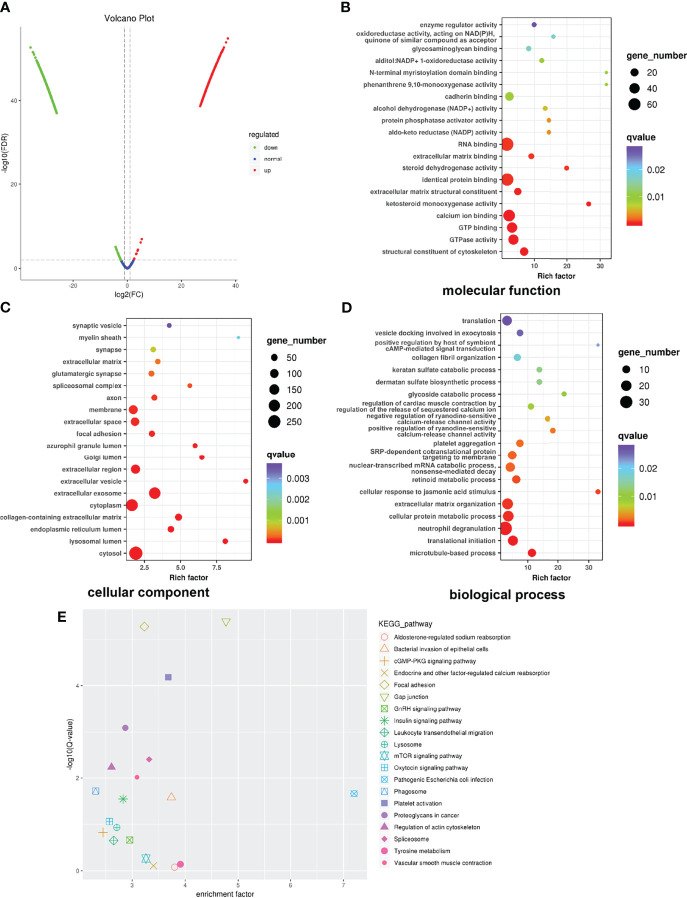
Global profile of exosomal proteins. **(A)** Volcano plot showing protein expression. **(B–D)** GO enrichment analysis of upregulated proteins exclusively identified in exosomes. The horizontal coordinate shows the enrichment factor, and the vertical coordinate represents the protein-enriched secondary GO function. The size and color of the dots indicate gene number and Q-value. **(E)** KEGG pathway enrichment of significantly upregulated proteins. GO, Gene Ontology; KEGG, Kyoto Encyclopedia of Genes and Genomes.

Proteomic analysis was performed to find global alterations of exosomal proteins and potential downstream effects on tumor progression. Protein expression volcano plots revealed that C_70_-EDA induced the upregulated expression of 468 proteins and the downregulated expression of 817 proteins in exosomes (**Figure 3A**). The significant protein difference in exosomes was visualized by hierarchical cluster analysis (**Figure S3**). As C_70_-EDA significantly increased exosomal proteins, the upregulated exosomal proteins are the most likely to regulate recipient cell functions. Therefore, Gene Ontology (GO) and Kyoto Encyclopedia of Genes and Genomes (KEGG) pathway enrichment analyses focused on significantly upregulated exosomal proteins (fold change >2) were performed to discover the biological effects of these proteins on recipient tumor cells. The enriched “molecular functions” terms were RNA binding, identical protein binding, calcium ion binding, GTP binding, and GTPase activity (**Figure 3B**). The “cellular components” terms included synaptic vesicle, membrane, focal adhesion, Golgi lumen, extracellular exosome, cytoplasm, endoplasmic reticulum lumen, lysosomal lumen, and cytosol (**Figure 3C**), which were associated with the exosome generation process. The regulated “biological processes” terms cover the translation, collagen fibril organization, extracellular matrix organization, translation initiation, and microtubule-based processes (**Figure 3D**). In the twenty most significantly enriched KEGG pathways, focal adhesion, gap junction, mTOR signaling pathway, and proteoglycans in cancer, are involved in tumor growth and metastasis (**Figure 3E**). Correspondingly, the WD40 domains, which were enriched in most of the identified genes, are essential subunits of multiprotein complexes involved in diverse cellular processes (**Figure S4**), including G protein-coupled receptor (GPCR) signaling, DNA damage sensing and repair, the ubiquitin-proteasome system (UPS), cell growth and division, epigenetic regulation of gene expression and chromatin organization, and the immune system (35).

### Promotion of Tumor Cell Proliferation by C_70_-EDA Exosomes

**Figure 4:**
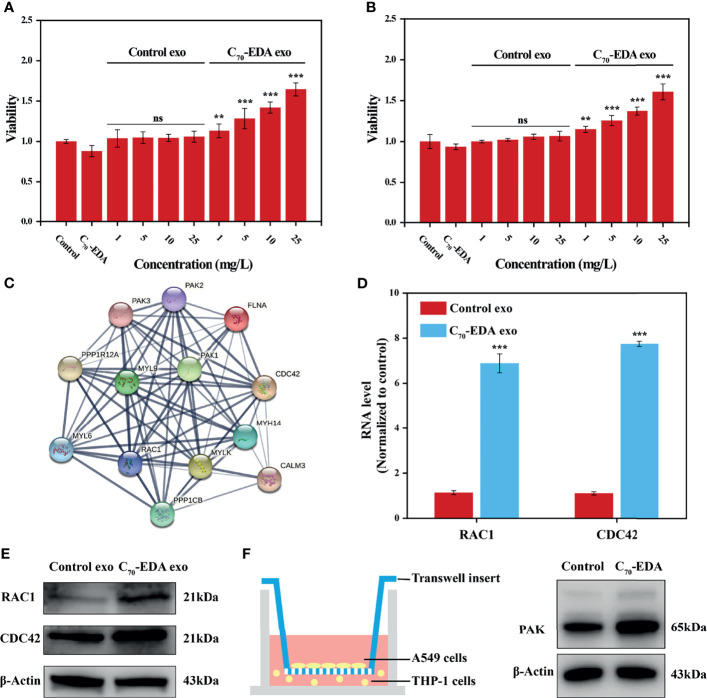
C_70_-EDA exosomes promote tumor cell proliferation. **(A, B)** The viability of A549 and U87-MG cells treated with exosomes at gradient protein concentrations for 24 h (n = 4). **(C)** Protein interaction network of upregulated proteins involved in the Rho GTPase/PAK pathway. **(D)** The mRNA levels of exosomal RAC1 and CDC42 were evaluated by RT–PCR (n = 4). **(E)** Expression of exosomal RAC1 and CDC42 proteins derived from THP-1 cells treated with C_70_-EDA (20 μM) for 48 h. **(F)** Left: schematic diagram showing the Transwell coculture with A549 cells and THP-1 cells pretreated with C_70_-EDA (20 μM) for 48 h. Right: expression of PAK protein in A549 cells. The data are reported as the means ± s.d., and n represents the number of biologically independent samples. Statistical significance was calculated *via* Student’s t-test. **p < 0.01 and ***p < 0.001. ns, non-significant.

C_70_-EDA induces the features of M2-like monocytes and increases the protein content in M2-like monocyte-derived exosomes. To investigate the effect of C_70_-EDA exosomes on tumor growth, A549 and U87-MG cells were treated with these exosomes. C_70_-EDA exosomes improved cell viability in a dose-dependence, but the control exosomes did not change the viability at protein concentrations up to 25 mg/L (**Figures 4A, B** **)**. A protein-protein interaction (PPI) network of the upregulated exosomal proteins was generated to identify the molecular mechanism by which C_70_-EDA exosomes enhance cancer cell proliferation. The exosomal proteins that had been increased by C_70_-EDA treatment of THP-1 cells were significantly enriched in the Rho GTPase-activated PAK Reactome pathway (**Figure S5**). Thirteen proteins are involved in Rho GTPase/PAK signaling: PPP1R12A, MYH14, MYLK, CDC42, RAC1, PAK1/2/3, PPP1CB, MYL6, MYL9, CALM3, and FLNA. PPI analysis revealed that all of these proteins were highly correlated (**Figure 4C**), with most association with cell proliferation and motility (**Table S2**). Both the mRNA and protein expression levels of RAC1 and CDC42 were significantly increased in the C_70_-EDA exosomes (**Figures 4D, E** **)**. RAC1 and CDC42 can activate PAK, thereby regulating cytoskeletal dynamics, the cell cycle, cell motility, and cell death and survival signaling processes (36). C_70_-EDA exosome-induced activation of the PAK pathway in tumor cells was investigated with a Transwell coculture model. THP-1 cells pretreated with C_70_-EDA (20 μM) for 24 h were placed in the lower chamber of Transwell inserts, and A549 cells were placed in the upper chamber. After coculture for 24 h, proteins in the A549 cells were extracted for Western blotting. C_70_-EDA exosomes upregulated the expression of the PAK protein in A549 cells (**Figure 4F**). These findings show that C_70_-EDA-treated M2-like monocyte-derived exosomes triggered the proliferation of tumor cells, probably through the activation of Rho GTPase/PAK signaling.

## Discussion

Monocyte and monocyte-derived TAMs are recruited throughout tumor development and progression and play critical roles in drug resistance (20, 37). Nanoparticles are thought to be rapidly internalized by TAMs and to serve as potential immunomodulators to activate TAMs (38). Nanosized hydroxylated fullerenes trigger immune responses, including upregulation of Th1 cytokine and downregulation of Th2 cytokine (29). Alanine-modified fullerenes reprogram TAMs, which switch from the M2 phenotype to the M1 phenotype and rebuild the TME, effectively inhibiting tumor growth (30). The surface charge of fullerene derivatives is a primary factor mediating the rate of their cellular uptake, subcellular location, and intracellular antioxidation behavior (39). Compared with the M1 polarization induced by negatively charged hydroxylated fullerenes, C_70_-EDA with positive surface charges was taken up by THP-1 cells and then triggered the acquisition of the M2-like phenotype (**Figure 1**). The opposite surface characteristics of aminated and hydroxylated fullerenes probably lead to the different remodeling effects on monocytes or macrophages.

Exosomes secreted by M2 TAMs can promote tumor growth and metastasis by delivering miRNA and/or protein cargoes to recipient tumor cells. C_70_-EDA increases the protein content in M2-like monocyte-derived exosomes by more than three-fold and is encapsulated in the exosomes, while the number and morphology of the exosomes are still maintained (**Figure 2**). Our previous work revealed that C_70_-EDA treatment increased the global protein levels in tumor cells *via* post-transcriptional regulation, which was realized by C_70_-EDA binding to mRNA-binding proteins and mRNA transport-associated proteins (28). Therefore, C_70_-EDA taken up by THP-1 cells probably enhances the translation process and thus accelerates intracellular protein synthesis, resulting in a corresponding increase in exosomal protein content. Moreover, positively charged C_70_-EDA can be easily encapsulated into exosomes through its electrostatic interactions with the exosomal membrane.

There were 468 upregulated proteins in C_70_-EDA exosomes, as revealed by proteomic profiling (**Figure 3A**). These proteins are mainly involved in RNA binding, translation, and organization of collagen fibrils and the extracellular matrix (**Figures 3B** **–D**), which are closely associated with cell proliferation and migration. C_70_-EDA exhibits specific affinity for RNA-binding proteins and myosin (27, 28), which probably contributes to the accumulation of these proteins in C_70_-EDA exosomes. A KEGG pathway enrichment analysis led to the identification of four classical signaling pathways regulating oncogenesis and metastasis: focal adhesion, gap junction, mTOR signaling, and proteoglycans in cancer (**Figure 3E**). Proteomic profiling of C_70_-EDA exosomes revealed their regulatory role in tumor progression, and C_70_-EDA exosomes indeed improved tumor cell proliferation, as predicted (**Figures 4A, B** **)**.

**Figure 5:**
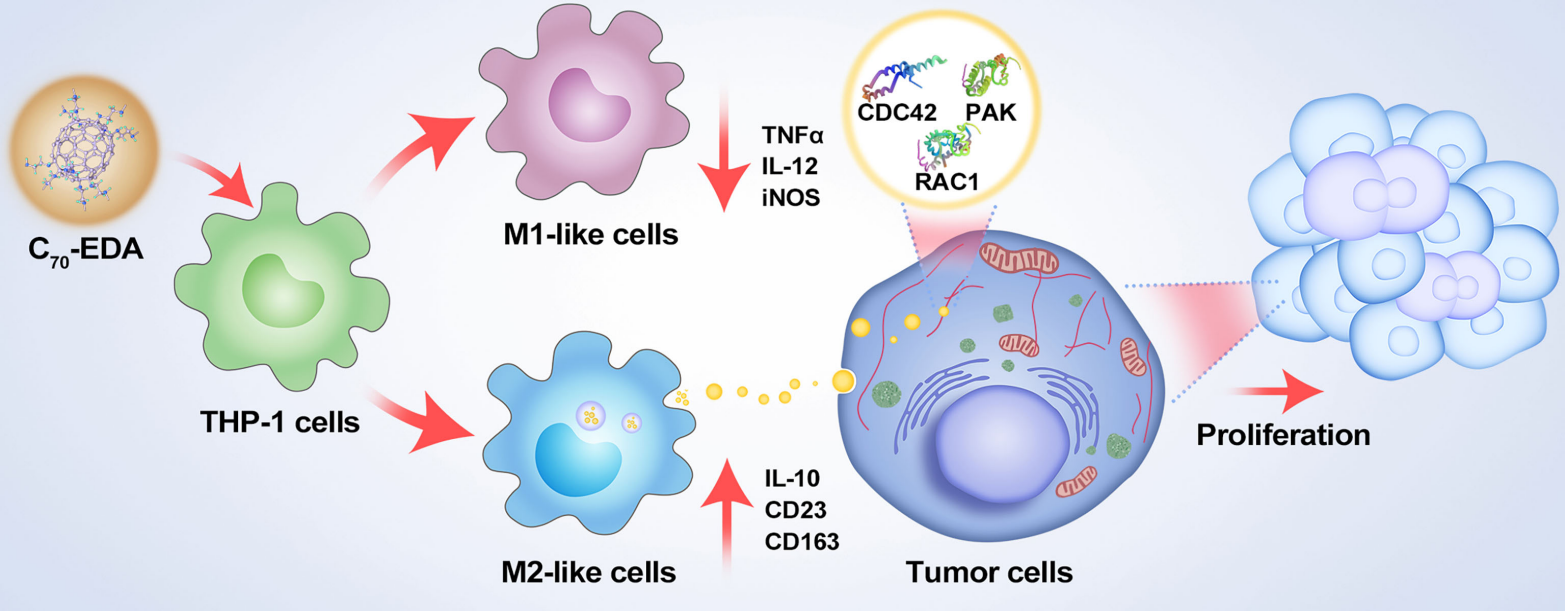
Schematic showing the mechanisms of potential resistance to antineoplastic therapy by C_70_-EDA triggering of THP-1 monocytes to the M2 phenotype and further promoting tumor proliferation by M2-like monocyte-derived exosomes.

The PPI network highlighted the Rho GTPase/PAK signaling pathway, which is likely critical for the C_70_-EDA exosome-induced increase in the proliferation of tumor cells. Rho family GTPases constitute a subgroup in the Ras superfamily of GTPases; this subgroup consists of 21 members, of which RAC1, RhoA, and CDC42 are the best-characterized. These three Rho members share significant amino acid sequence homology. However, each member exerts different biological effects on the actin cytoskeleton (40) and is involved in cell cycle control, epithelial cell polarity, cell migration, cell survival, and angiogenesis. The (GEF) Asef2 has been shown to increase RAC and CDC42 expression, resulting in enhanced cancer cell migration and metastasis (41). The essential downstream regulator of RAC1 and CDC42 is PAK, which plays vital roles in cancer initiation, growth, angiogenesis, immunity, metabolism, metastasis, and drug resistance (42–44). Upregulation of PAK expression by RAC/CDC42 induces the formation of lamellipodia, filopodia, membrane folds, and stress fibers, and the remodeling of focal adhesion complexes (45). PAK regulates cancer cell growth *via* several signaling pathways, including the WNT/β-catenin, EGFR/HER2/MAPK, and PI3K/AKT pathways (46). C_70_-EDA could induce G0/G1 cell cycle arrest to abrogate cancer cell proliferation (28). However, C_70_-EDA exosomes with excessive RAC1 and CDC42 expression promoted the proliferation of recipient A549 and U87-MG cells, probably because they activated the PAK signaling pathway. This outcome indicates that the antineoplastic effect of C_70_-EDA *via* inducing cell cycle arrest may be attenuated once C_70_-EDA is internalized by monocytes (**Figure 5**).

In conclusion, this study revealed that antineoplastic aminated fullerenes induced the features of M2-like monocytes and significantly increased the protein content in exosomes secreted by M2-like monocytes. Notably, M2-like monocyte-derived exosomes boosted the proliferation of recipient tumor cells probably by activating the Rho GTPase/PAK pathway. C_70_-EDA triggered the M2-like state of monocytes and M2-like monocyte-derived exosomes may contribute to a pro-tumor immunity, indicating that a combination of aminated fullerenes and inhibitors of M2 phenotype acquisition/switching or PAK pathway activation may lead to synergistic antineoplastic effects.

## Data Availability Statement

The original contributions presented in the study are included in the article/**Supplementary Material**. Further inquiries can be directed to the corresponding authors.

## Author Contributions

JH, JL, and CW designed the research. JH, JL, and YL synthesized and characterized the materials. JH and SY performed the cell experiments and data analysis. JH, WZ, JL, and CW prepared the paper. All authors have read and approved the article.

## Conflict of Interest

The authors declare that the research was conducted in the absence of any commercial or financial relationships that could be construed as a potential conflict of interest.

## Publisher’s Note

All claims expressed in this article are solely those of the authors and do not necessarily represent those of their affiliated organizations, or those of the publisher, the editors and the reviewers. Any product that may be evaluated in this article, or claim that may be made by its manufacturer, is not guaranteed or endorsed by the publisher.

## References

[1] Kalluri R Lebleu VS. The Biology, Function, and Biomedical Applications of Exosomes. Science (2020) 367(6478):eaau6977. doi: 10.1126/science.aau6977 32029601 PMC7717626

[2] Théry C Witwer KW Aikawa E Alcaraz MJ Anderson JD Andriantsitohaina R. Minimal Information for Studies of Extracellular Vesicles 2018 (MISEV2018): A Position Statement of the International Society for Extracellular Vesicles and Update of the MISEV2014 Guidelines. J Extracell Vesicles (2018) 7(1):1535750. doi: 10.1080/20013078.2018.1535750 30637094 PMC6322352

[3] Urbanelli L Magini A Buratta S Brozzi A Sagini K Polchi A. Signaling Pathways in Exosomes Biogenesis, Secretion and Fate. Genes (2013) 4(2):152–70. doi: 10.3390/genes4020152 PMC3899971 24705158

[4] Rao L Wu L Liu Z Tian R Yu G Zhou Z. Hybrid Cellular Membrane Nanovesicles Amplify Macrophage Immune Responses Against Cancer Recurrence and Metastasis. Nat Commun (2020) 11(1):1–13. doi: 10.1038/s41467-020-18626-y 32999291 PMC7527506

[5] Meng QF Zhao Y Dong C Liu L Pan Y Lai J. Genetically Programmable Fusion Cellular Vesicles for Cancer Immunotherapy. Angewandte Chemie Int Ed (2021) 60(50):26320–6. doi: 10.1002/anie.202108342 34661332

[6] Sung S Kim J Jung Y. Liver-Derived Exosomes and Their Implications in Liver Pathobiology. Int J Mol Sci (2018) 19(12):3715. doi: 10.3390/ijms19123715 PMC6320937 30469540

[7] Nikfarjam S Rezaie J Kashanchi F Jafari R. Dexosomes as a Cell-Free Vaccine for Cancer Immunotherapy. J Exp Clin Cancer Res (2020) 39(1):1–20. doi: 10.1186/s13046-020-01781-x 33228747 PMC7686678

[8] Babaei M Rezaie J. Application of Stem Cell-Derived Exosomes in Ischemic Diseases: Opportunity and Limitations. J Trans Med (2021) 19(1):1–11. doi: 10.1186/s12967-021-02863-w PMC8106139 33964940

[9] Jabbari N Nawaz M Rezaie J. Bystander Effects of Ionizing Radiation: Conditioned Media From X-Ray Irradiated MCF-7 Cells Increases the Angiogenic Ability of Endothelial Cells. Cell Commun Signal (2019) 17(1):1–12. doi: 10.1186/s12964-019-0474-8 31842899 PMC6912994

[10] Kreger B Johansen E Cerione R Antonyak M. The Enrichment of Survivin in Exosomes From Breast Cancer Cells Treated With Paclitaxel Promotes Cell Survival and Chemoresistance. Cancers (2016) 8(12):111. doi: 10.3390/cancers8120111 PMC5187509 27941677

[11] Chen W-X Cai Y-Q Lv M-M Chen L Zhong S-L Ma T-F. Exosomes From Docetaxel-Resistant Breast Cancer Cells Alter Chemosensitivity by Delivering microRNAs. Tumor Biol (2014) 35(10):9649–59. doi: 10.1007/s13277-014-2242-0 24969560

[12] Li XJ Zhao JR Tang JH Yu Q. Exosomal MicroRNA MiR-1246 Promotes Cell Proliferation, Invasion and Drug Resistance by Targeting CCNG2 in Breast Cancer. Cell Physiol Biochem (2017) 44(5):1741–8. doi: 10.1159/000485780 29216623

[13] Chen G Huang AC Zhang W Zhang G Wu M Xu W. Exosomal PD-L1 Contributes to Immunosuppression and is Associated With Anti-PD-1 Response. Nature (2018) 560(7718):382–6. doi: 10.1038/s41586-018-0392-8 PMC6095740 30089911

[14] Steinbichler TB Dudás J Skvortsov S Ganswindt U Riechelmann H Skvortsova I-I. Therapy Resistance Mediated by Exosomes. Mol Cancer (2019) 18(1):1–11. doi: 10.1186/s12943-019-0970-x 30925921 PMC6441190

[15] Dong X Bai X Ni J Zhang H Duan W Graham P. Exosomes and Breast Cancer Drug Resistance. Cell Death Dis (2020) 11(11):1–14. doi: 10.1038/s41419-020-03189-z 33203834 PMC7673022

[16] Olingy CE Dinh HQ Hedrick CC. Monocyte Heterogeneity and Functions in Cancer. J Leukocyte Biol (2019) 106(2):309–22. doi: 10.1002/jlb.4ri0818-311r PMC6658332 30776148

[17] Martinez FO Sica A Mantovani A Locati M. Macrophage Activation and Polarization. Front Biosci-Landmark (2008) 13(2):453–61. doi: 10.2741/2692 17981560

[18] Van Den Heuvel MM Tensen CP Van As JH Van Den Berg TK Fluitsma DM Dijkstra CD. Regulation of CD163 on Human Macrophages: Cross-Linking of CD163 Induces Signaling and Activation. J Leukocyte Biol (1999) 66(5):858–66. doi: 10.1002/jlb.66.5.858 10577520

[19] Han C Zhang C Wang H Zhao L. Exosome-Mediated Communication Between Tumor Cells and Tumor-Associated Macrophages: Implications for Tumor Microenvironment. OncoImmunology (2021) 10(1):1887552. doi: 10.1080/2162402x.2021.1887552 33680573 PMC7901554

[20] Zheng P Luo Q Wang W Li J Wang T Wang P. Tumor-Associated Macrophages-Derived Exosomes Promote the Migration of Gastric Cancer Cells by Transfer of Functional Apolipoprotein E. Cell Death Dis (2018) 9(4):1–14. doi: 10.1038/s41419-018-0465-5 29567987 PMC5864742

[21] Azambuja JH Ludwig N Yerneni SS Braganhol E Whiteside TL. Arginase-1+ Exosomes From Reprogrammed Macrophages Promote Glioblastoma Progression. Int J Mol Sci (2020) 21(11):3990. doi: 10.3390/ijms21113990 PMC7312363 32498400

[22] Wu J Gao W Tang Q Yu Y You W Wu Z. M2 Macrophage–Derived Exosomes Facilitate HCC Metastasis by Transferring α M β 2 Integrin to Tumor Cells. Hepatology (2021) 73(4):1365–80. doi: 10.1002/hep.31432 PMC8360085 32594528

[23] Kang S-G Zhou G Yang P Liu Y Sun B Huynh T. Molecular Mechanism of Pancreatic Tumor Metastasis Inhibition by Gd@C82(OH)22 and its Implication for *De Novo* Design of Nanomedicine. Proc Natl Acad Sci (2012) 109(38):15431–6. doi: 10.1073/pnas.1204600109 PMC3458392 22949663

[24] Liu Y Chen C Qian P Lu X Sun B Zhang X. Gd-Metallofullerenol Nanomaterial as non-Toxic Breast Cancer Stem Cell-Specific Inhibitor. Nat Commun (2015) 6(5988):1–18. doi: 10.1038/ncomms6988 PMC4354030 25612916

[25] Zhen M Shu C Li J Zhang G Wang T Luo Y. A Highly Efficient and Tumor Vascular-Targeting Therapeutic Technique With Size-Expansible Gadofullerene Nanocrystals. Sci China Mater (2015) 58(10):799–810. doi: 10.1007/s40843-015-0089-3

[26] Li J Chen L Su H Yan L Gu Z Chen Z. The Pharmaceutical Multi-Activity of Metallofullerenol Invigorates Cancer Therapy. Nanoscale (2019) 11(31):14528–39. doi: 10.1039/c9nr04129j 31364651

[27] Zhou W Huo J Yang Y Zhang X Li S Zhao C. Aminated Fullerene Abrogates Cancer Cell Migration by Directly Targeting Myosin Heavy Chain 9. ACS Appl Mater Interfaces (2020) 12(51):56862–73. doi: 10.1021/acsami.0c18785 33305958

[28] Zhang X Zhou W Liu Y Jin L Huo J Yang Y. Nanosize Aminated Fullerene for Autophagic Flux Activation and G0/G1 Phase Arrest in Cancer Cells *via* Post-Transcriptional Regulation. Nano Res (2021) 14(10):1–10. doi: 10.1007/s12274-021-3866-1

[29] Liu Y Jiao F Qiu Y Li W Lao F Zhou G. The Effect of Gd@C-82(OH)(22) Nanoparticles on the Release of Th1/Th2 Cytokines and Induction of TNF-Alpha Mediated Cellular Immunity. Biomaterials (2009) 30(23-24):3934–45. doi: 10.1016/j.biomaterials.2009.04.001 19403166

[30] Li L Zhen M Wang H Sun Z Jia W Zhao Z. Functional Gadofullerene Nanoparticles Trigger Robust Cancer Immunotherapy Based on Rebuilding an Immunosuppressive Tumor Microenvironment. Nano Lett (2020) 20(6):4487–96. doi: 10.1021/acs.nanolett.0c01287 32407113

[31] Kim H Cha J Jang M Kim P. Hyaluronic Acid-Based Extracellular Matrix Triggers Spontaneous M2-Like Polarity of Monocyte/Macrophage. Biomater Sci (2019) 7(6):2264–71. doi: 10.1039/c9bm00155g 30849138

[32] Sawa-Wejksza K Dudek A Lemieszek M Kaławaj K Kandefer-Szerszeń M. Colon Cancer–Derived Conditioned Medium Induces Differentiation of THP-1 Monocytes Into a Mixed Population of M1/M2 Cells. Tumor Biol (2018) 40(9):101042831879788. doi: 10.1177/1010428318797880 30183516

[33] Zhang B Cao M He Y Liu Y Zhang G Yang C. Combination of Plasma HA and Circulating M2-Like Monocytes may Serve as a Diagnostic Marker for Breast Cancer. J Cancer (2017) 8(17):3522–30. doi: 10.7150/jca.20227 PMC5687167 29151937

[34] Liu Y-C Zou X-B Chai Y-F Yao Y-M. Macrophage Polarization in Inflammatory Diseases. Int J Biol Sci (2014) 10(5):520–9. doi: 10.7150/ijbs.8879 PMC4046879 24910531

[35] Schapira M Tyers M Torrent M Arrowsmith CH. WD40 Repeat Domain Proteins: A Novel Target Class? Nat Rev Drug Discov (2017) 16(11):773–86. doi: 10.1038/nrd.2017.179 PMC5975957 29026209

[36] Chan PM Manser E. PAKs in Human Disease. Prog Mol Biol Transl Sci (2012) 106:171–87. doi: 10.1016/B978-0-12-396456-4.00011-0 22340718

[37] Qian BZ Pollard JW. Macrophage Diversity Enhances Tumor Progression and Metastasis. Cell (2010) 141(1):39–51. doi: 10.1016/j.cell.2010.03.014 20371344 PMC4994190

[38] Weissleder R Nahrendorf M Pittet MJ. Imaging Macrophages With Nanoparticles. Nat Mater (2014) 13(2):125–38. doi: 10.1038/nmat3780 24452356

[39] Ma H Zhang X Yang Y Li S Huo J Liu Y. Cellular Uptake, Organelle Enrichment, and *In Vitro* Antioxidation of Fullerene Derivatives, Mediated by Surface Charge. Langmuir (2021) 37(8):2740–8. doi: 10.1021/acs.langmuir.0c03483 33586439

[40] Grise F Bidaud A Moreau V. Rho GTPases in Hepatocellular Carcinoma. Biochim Biophys Acta (BBA) - Rev Cancer (2009) 1795(2):137–51. doi: 10.1016/j.bbcan.2008.12.003 19162129

[41] Bristow JM Sellers MH Majumdar D Anderson B Hu L Webb DJ. The Rho-Family GEF Asef2 Activates Rac to Modulate Adhesion and Actin Dynamics and Thereby Regulate Cell Migration. J Cell Sci (2009) 122(24):4535–46. doi: 10.1242/jcs.053728 PMC2787464 19934221

[42] Venu A Archana B Kanumuri R Vuttaradhi VK D'Cruze L Murugan S. Clinical Evaluation of P21 Activated Kinase 1 (PAK1) Activation in Gliomas and Its Effect on Cell Proliferation. Cancer Invest (2021) 39(1):98–113. doi: 10.1080/07357907.2020.1858097 33251876

[43] Bautista L Knippler CM Ringel MD. P21-Activated Kinases in Thyroid Cancer. Endocrinology (2020) 161(8):1–11. doi: 10.1210/endocr/bqaa105 PMC7417880 32609833

[44] Huang H Xue Q Du X Cui J Wang J Cheng D. P21-Activated Kinase 4 Promotes the Progression of Esophageal Squamous Cell Carcinoma by Targeting LASP1. Mol Carcinog (2021) 60(1):38–50. doi: 10.1002/mc.23269 PMC7756368 33289209

[45] Bokoch GM. Biology of the P21-Activated Kinases. Annu Rev Biochem (2003) 72(1):743–81. doi: 10.1146/annurev.biochem.72.121801.161742 12676796

[46] Liu H Liu K Dong Z. The Role of P21-Activated Kinases in Cancer and Beyond: Where Are We Heading? Front Cell Dev Biol (2021) 9:641381. doi: 10.3389/fcell.2021.641381 33796531 PMC8007885

