# Supplementary material for: Potential Resistance to Antineoplastic Aminated Fullerenes Mediated by M2-Like Monocyte-Derived Exosomes
Source: Front Oncol. 2022 Mar 31;12:779939. doi: 10.3389/fonc.2022.779939 (PMC9009388; doi:10.3389/fonc.2022.779939)
Supplement: Supplementary file 1 [file DataSheet_1.docx]

**Supporting Information**

**Potential Resistance to Antineoplastic Aminated Fullerenes Mediated by M2-like Monocyte-Derived Exosomes**

Jiawei Huo^a,b^, Wei Zhou^a^, Yang Liu^a,b^, Sifen Yang^a^, Jie Li^a,*^, Chunru Wang^a,b*^

[a] Beijing National Research Center for Molecular Sciences

Key Laboratory of Molecular Nanostructure and Nanotechnology, Institute of Chemistry, Chinese Academy of Science, Beijing 100190, China

[b] University of Chinese Academy of Sciences

E-mail: crwang@iccas.ac.cn, lijie24@iccas.ac.cn

*   To whom correspondence should be addressed

**KEYWORDS**: C_70_-EDA, M2-like monocyte-derived exosome, proteomics, Rho GTPase/PAK signaling, tumor proliferation

Results

**Table S1** | List of primer sequences.

| **Name** | **Sequence (5’ to 3’)** |
| --- | --- |
| TNFα Forward | CTTCTGCCTGCTGCACTTTG |
| TNFα Reverse | GGCCAGAGGGCTGATTAGAGA |
| GAPDH Forward | GGAGCGAGATCCCTCCAAAAT |
| GAPDH Reverse | GGCTGTTGTCATACTTCTCATGG |
| IL-12 Forward | ATTCGCTCCTGCTGCTTCACA |
| IL-12 Reverse | CGTCCAGAATAATTCTTGGCCTC |
| iNOS Forward | AGCGGGATGACTTTCCAAGA |
| iNOS Reverse | GGACCCCAGGCAAGATTTG |
| CXCL9 Forward | CATCTTGCTGGTTCTGATTGGA |
| CXCL9 Reverse | GTCCCTTGGTTGGTGCT |
| CCR7 Forward | GCTGGTGGTGGCTCTCCTT |
| CCR7 Reverse | GTAATCGTCCGTGACCTCATCTT |
| IL-10 Forward | GAGGCTACGGCGCTGTCA |
| IL-10 Reverse | TCCACGGCCTTGCTCTTG |
| Arg2 Forward | GACAAGCAACAAACCCTTGATG |
| Arg2 Reverse | AGGACAAACTGCTCTGCCAATT |
| CD23 Forward | CCCGGAACGTCTCTCAAGTTT |
| CD23 Reverse | TCAGCTCGAAGTTCCTCCAGTT |
| CD163 Forward | CAGTGCAGAAAACCCCACAA |
| CD163 Reverse | AAAGGATGACTGACGGGATGA |
| RAC1 Forward | ATGTCCGTGCAAAGTGGTATC |
| RAC1 Reverse | CTCGGATCGCTTCGTCAAACA |
| CDC42 Forward | CCATCGGAATATGTACCGACTG |
| CDC42 Reverse | CTCAGCGGTCGTAATCTGTCA |


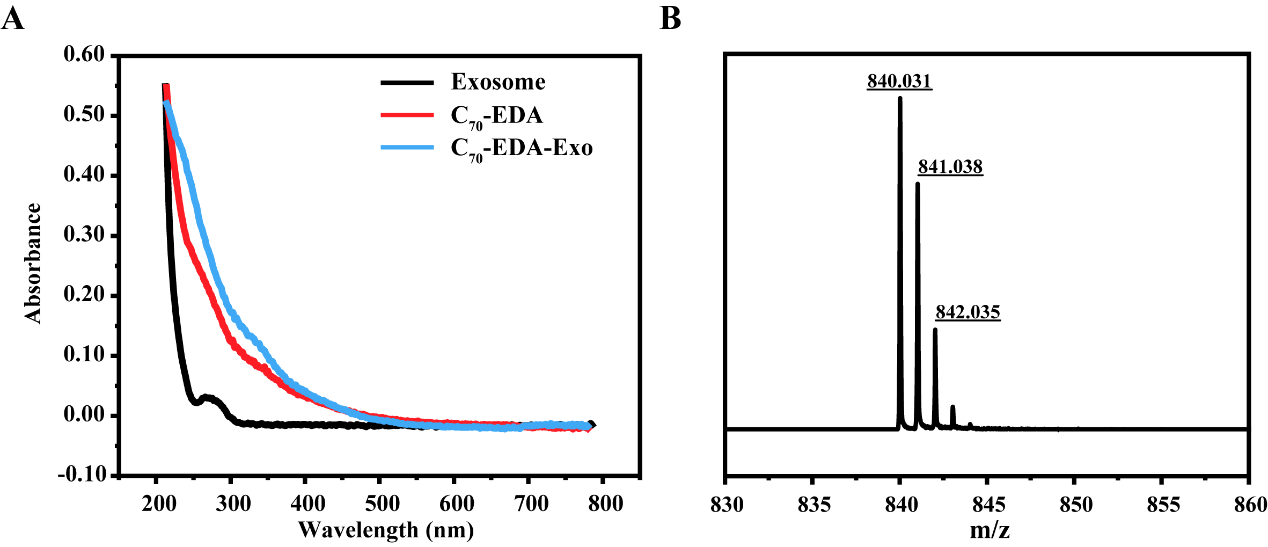


**Figure S1** | C_70_-EDA enters inside the exosomes secreted by THP-1 cells. (A) UV spectra of C_70_-EDA, control exosomes, and C_70_-EDA exosomes. (B) mass spectra of C_70_-EDA exosomes.


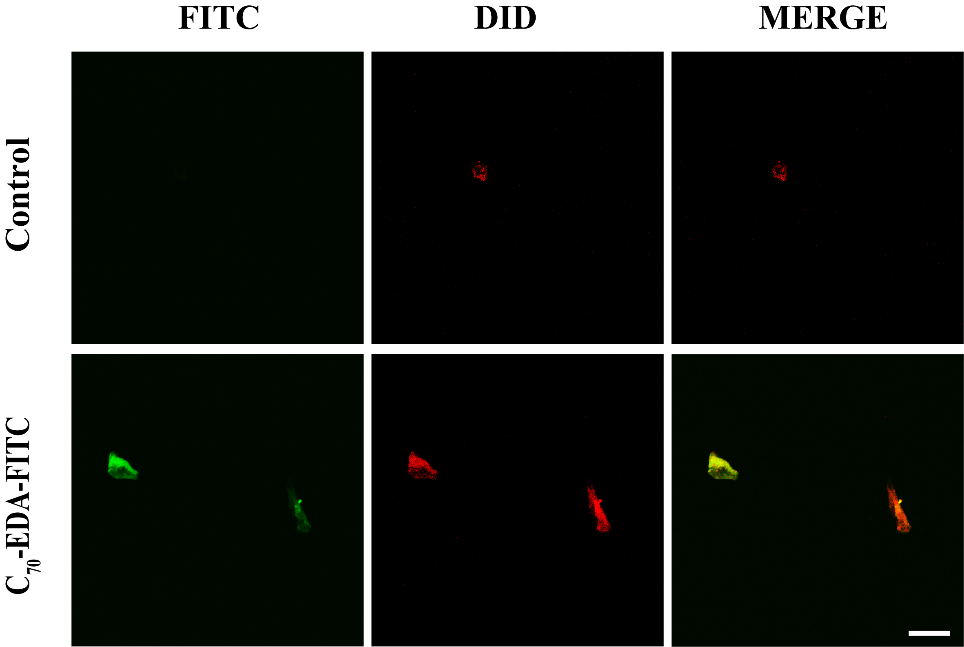


**Figure S2** | Fluorescence images of THP-1 cells treated with C_70_-EDA-FTIC (green) at 20 μM for 24 h and followed by staining with DID (red). The scale bar is 30 μm.


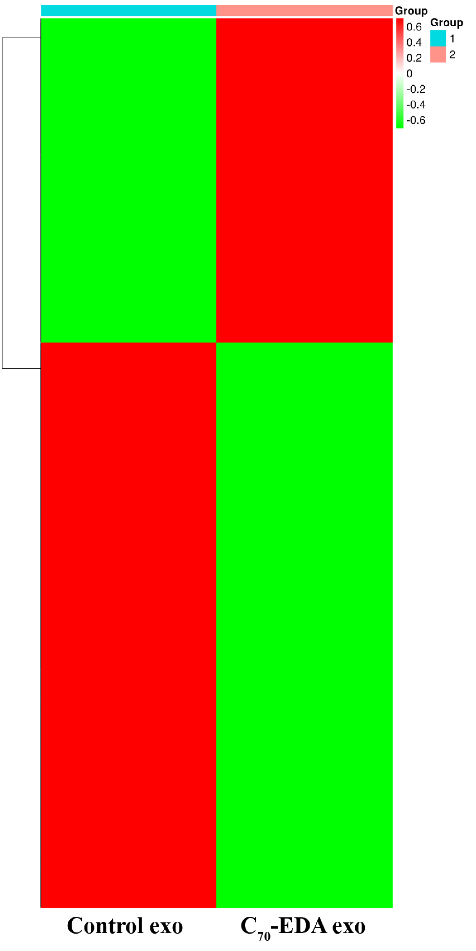


**Figure S3** | Clustering diagram of differentially expressed proteins. Columns represent different samples, and rows represent different proteins clustered with log10 (LFQ+1) values.


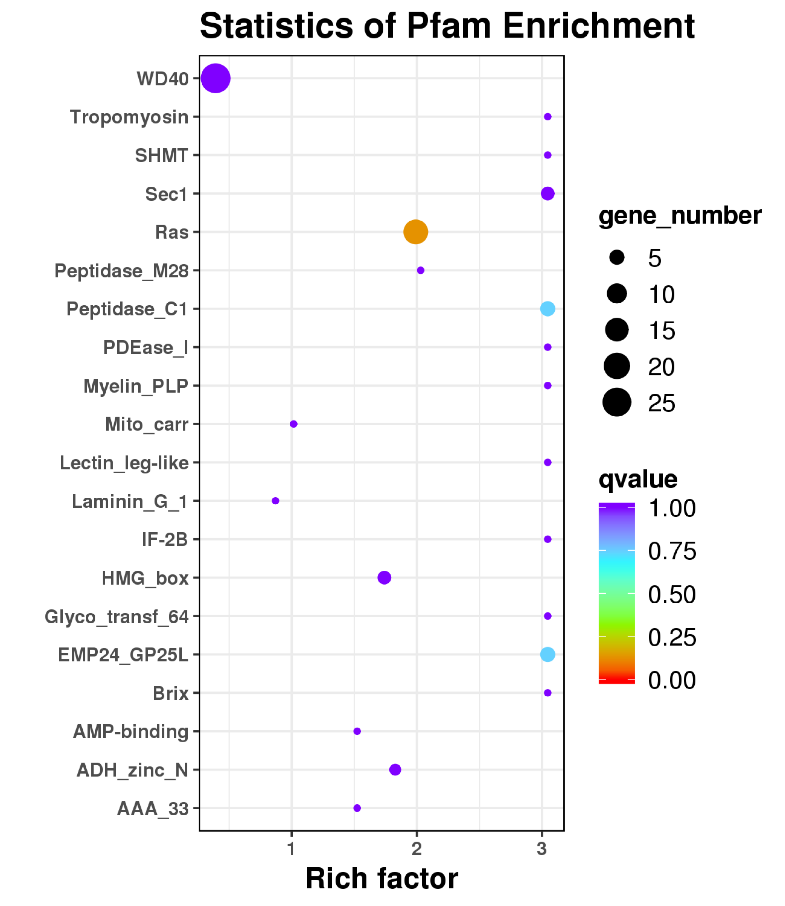


**Figure S4** | Bubble plots of structural domains enriched for differential proteins. The horizontal coordinate is the enrichment factor; the vertical coordinate is the name of the structural domain. The size of the dot indicates the number of proteins annotated to this structural domain. The color indicates the Q value of the significance P-value corrected by multiple hypothesis testing.


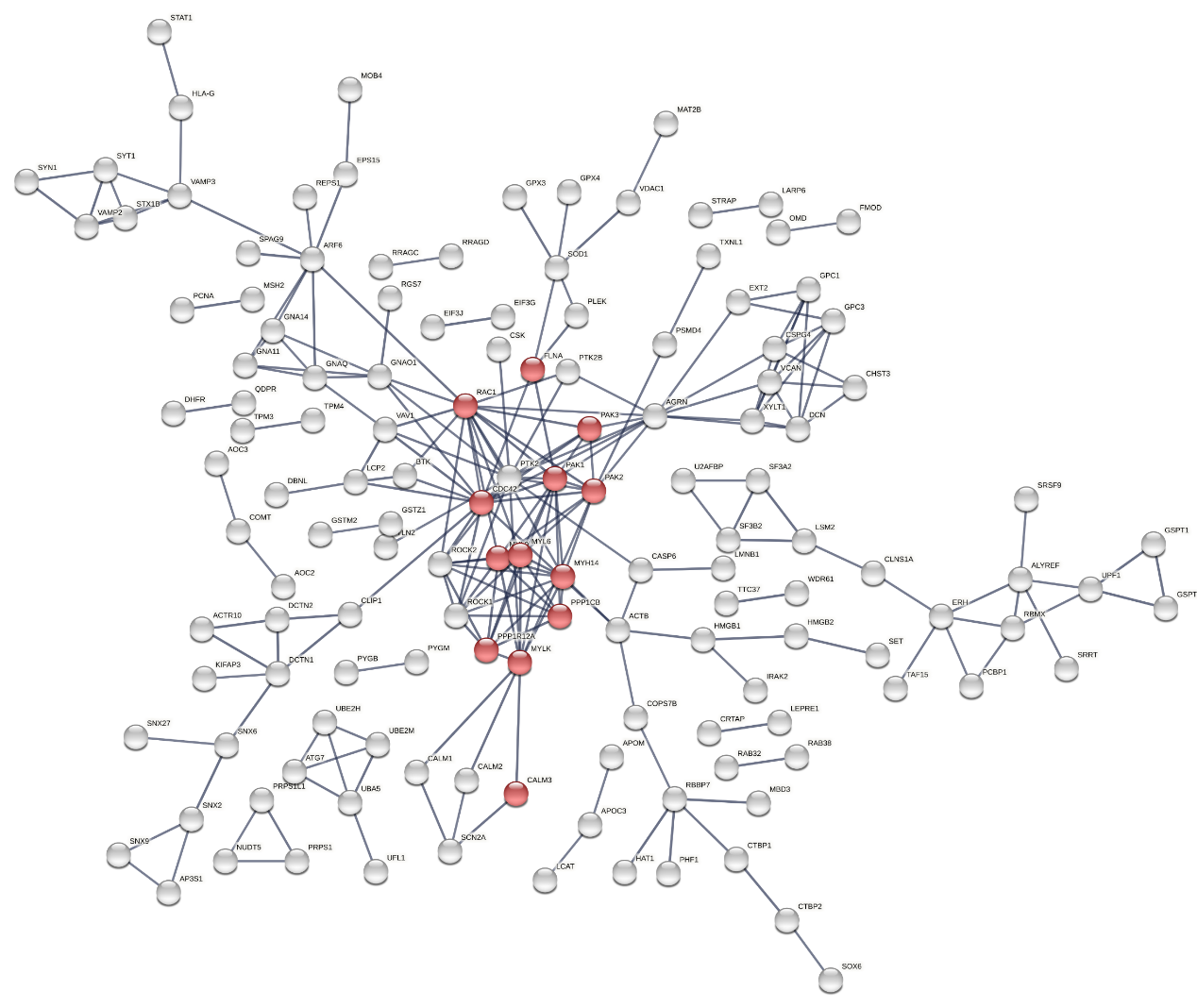


**Figure S5** | Interaction network of significantly up-regulated proteins, and the red nodes indicate proteins involved in the “RHO GTPases activate PAKs” Reactome pathway.

**Table S2** | Functional annotation of proteins enriched in the “RHO GTPases activate PAKs” Reactome pathway.

| **#ID** | **Function** |
| --- | --- |
| PPP1R12A | Key regulator of protein phosphatase 1C (PPP1C). Mediates binding to myosin. |
| MYH14 | Cellular myosin appears to play a role in cytokinesis, cell shape, and specialized functions such as secretion and capping. |
| MYLK | Involved in the inflammatory response, cell motility, morphology, and other activities relevant to asthma. |
| CDC42 | Involved in epithelial cell polarization processes. Regulates cell migration. |
| RAC1 | In its active state, it binds to a variety of effector proteins to regulate cellular responses. |
| PAK1; PAK2; PAK3 | Protein kinase is involved in intracellular signaling pathways that play an essential role in cytoskeleton dynamics, cell adhesion, migration, proliferation, apoptosis, mitosis, and vesicle-mediated transport processes. |
| PPP1CB | Protein phosphatase (PP1) is essential for cell division. |
| MYL6 | The regulatory light chain of myosin. |
| MYL9 | Myosin regulatory subunit plays an essential role in regulating both smooth muscle and nonmuscle cell contractile activity. |
| CALM3 | Calmodulin mediates the control of many enzymes, ion channels, aquaporins, and other proteins through calcium-binding. |
| FLNA | Promotes orthogonal branching of actin filaments and links actin filaments to membrane glycoproteins. |
